# Axillary Block as the Sole Anesthetic for Peripherally Inserted Central Catheter Placement in an Infant with Goldenhar Syndrome

**DOI:** 10.1155/2013/956807

**Published:** 2013-11-25

**Authors:** Ma. Carmen Bernardo-Ocampo

**Affiliations:** Seattle Children's Hospital, Seattle, WA 98105, USA

## Abstract

The use of peripheral nerve block as the sole anesthetic in infants is not very common. Studies have demonstrated that ultrasound guided (USG) peripheral nerve block is associated with higher overall success rate when compared with nerve stimulation (Rubin et al., 2009, and Gelfand et al., 2011). Described below is a medically complex infant who had an USG axillary brachial plexus block for peripherally inserted central catheter (PICC) placement.

## 1. Case Description

**Figure 1:**
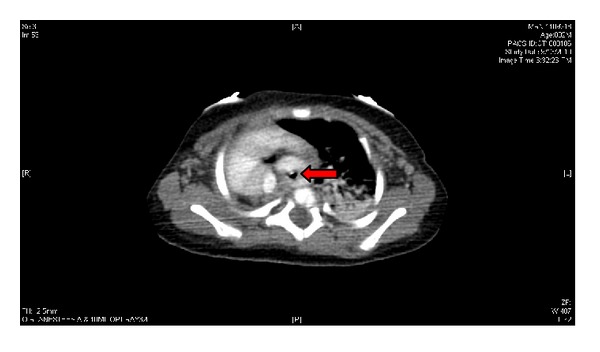
Tracheal compression, supine.

**Figure 2:**
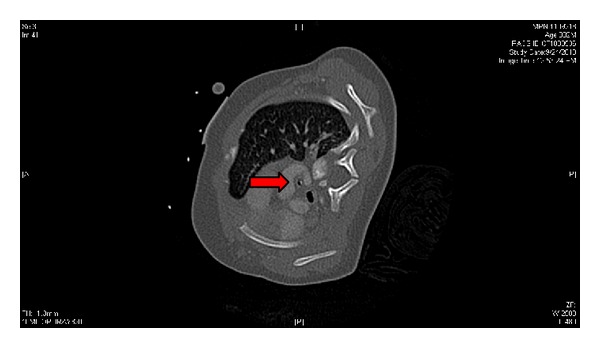
Further airway compression, RLDP.

The patient was a 2-month-old, 4.8 kg, full-term female with Goldenhar syndrome. Her congenital anomalies included right-sided cleft lip and palate, severe right lung hypoplasia with hypoplastic right pulmonary arteries and veins, dextrocardia with mediastinal shift, left-sided aortic arch with aberrant right subclavian artery creating tracheal compression, severe distal tracheomalacia, hypoplastic temporomandibular joint, micrognathia, right-sided microtia, right renal agenesis, left solitary kidney with duplicated collecting system, transverse liver, asplenia, rib anomalies, and scoliosis. Her other presenting problem was significant gastroesophageal reflux for which she had been G-tube feed dependent. Pertinent history was cardiac arrest in the operating room when she was positioned right side down for aortopexy (which was aborted at that time) and two episodes of profound bradycardia with hypotension in the intensive care unit when she was turned to her right. Evaluations to look for the etiology of the above episodes included echocardiogram and CT angiography. The echocardiogram on right lateral decubitus position (RLDP) showed no vascular deformation/compression. The CT angiography, done with the patient partially on RLDP, showed worsening of the tracheal narrowing from the previous 3 mm to 1 mm (Figures 1 and 2). 

This patient was referred to the Radiology and Anesthesiology Departments for PICC placement. At the PICU, she was lying on a wedge with her body slightly tilted to the left, breathing spontaneously with oxygen per nasal cannula, with suprasternal retractions (apparently her baseline), with NG tube attached to a continuous suction, and hemodynamically stable.

She was transported with the standard monitors to the interventional radiology suite on the same position as she was at the PICU. The wedge and slight left body tilt was maintained for positioning on the procedure table. Oxygen per nasal cannula and NG tube to suction were continued. After scanning both arms, the interventional radiologist decided that a vein from the right arm would be the most suitable. The team agreed to an USG axillary block for analgesia. After doing the preoperative checklist, the right arm was abducted and the axillary area was aseptically prepared. Using a 13–6 MHz, 25 mm transducer, the USG axillary block was done with a 25 g hypodermic needle and ropivacaine 0.5% 0.8 mL. The patient was given a pacifier dipped in dextrose water. She was comfortable and did not react to the skin prep and needle puncture. No supplemental medication was necessary throughout the procedure. The central venous catheter placement took 28 minutes. She was transported back to the PICU awake and with stable vital signs. Six hours after the axillary block, she was moving both upper extremities and had no residual right arm weakness. No hematoma or bruising in the axilla was noted.

## 2. Discussion

There are several anesthetic options for PICC placement in pediatric patients. The most common of these is the use of general anesthetic agents. Studies suggesting neurotoxicity of general anesthetic agents in the developing brain of animals and humans [1, 2] make other options for anesthetizing this patient population more enticing. Low-dose narcotic infusion in combination with sucrose and nonnutritive sucking has been found to be effective in decreasing pain and distress in preterm infants [3]. However, it did not make PICC placement easier and faster [3]. Application of topical anesthetic cream has been shown to lessen the changes in vital signs during PICC placement in very low birth weight infants [4]. A recent article reported that there was no significant difference in the Neonatal Infant Pain Score (NIPS) of preterm neonates who had topical anesthetic during PICC placement when compared with glucose and placebo [5]. An axillary approach to the brachial plexus using anatomic landmarks has been demonstrated to be effective for PICC placement in small infants [6]. Ultrasound guidance has been shown to increase block placement success rate, shorten block performance and onset times, and require lower volume of local anesthetic in children [7].

Patients with Goldenhar syndrome present with oral, tracheal, pulmonary, cardiac, and central nervous system abnormalities that may significantly influence the choice of anesthesia. The potential for a difficult airway in these patients is a prime consideration. For a medically complex patient with a potentially difficult airway and a very high risk of aspiration like our patient, a technique that would avoid or at least minimize these problems and at the same time allow for a safe and quick PICC placement is most appropriate. This case demonstrates that USG axillary block, without general anesthesia, can be a safe and effective anesthetic technique for PICC placement in critically ill infants.
